# Supplementary material for: circNDUFB2 inhibits non-small cell lung cancer progression via destabilizing IGF2BPs and activating anti-tumor immunity
Source: Nat Commun. 2021 Jan 12;12:295. doi: 10.1038/s41467-020-20527-z (PMC7804955; doi:10.1038/s41467-020-20527-z)
Supplement: Supplementary file 1 — Supplementary Information [file 41467_2020_20527_MOESM1_ESM.pdf]

Supplementary Information

# *circNDUFB2* inhibits non-small cell lung cancer progression via destabilizing IGF2BPs and activating anti-tumor immunity

Botai Li,[1] Lili Zhu,[1] Chunlai Lu,[1] Liyan Jiang[*] and Wenxin Qin[*]

[1]These authors contributed equally

*Corresponding authors

Supplementary Figure 1

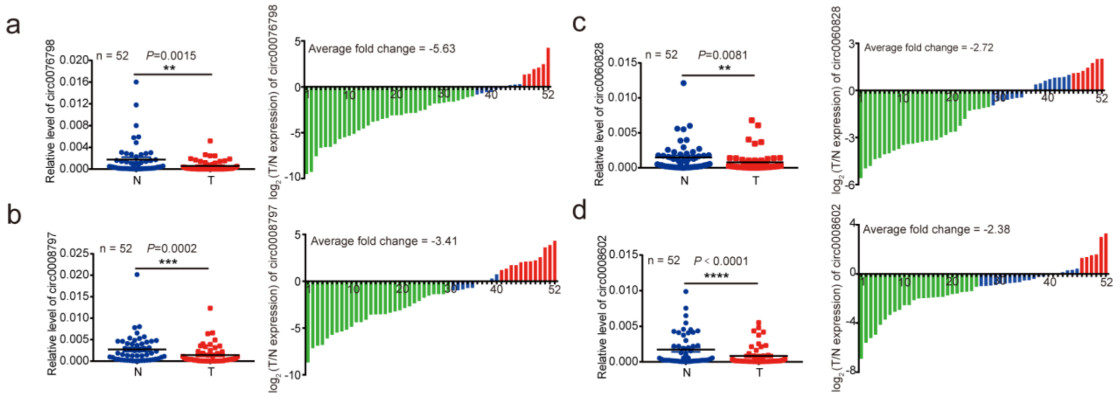


Other four circRNAs remarkably downregulated in NSCLC tissues.

(a-d) Expression levels of the indicated circRNAs in additional 52 paired samples of NSCLC. *GAPDH* was used as the loading control. n=52 biologically independent paired tissues of NSCLC. P values are calculated by paired two-sided t-test in a-d.

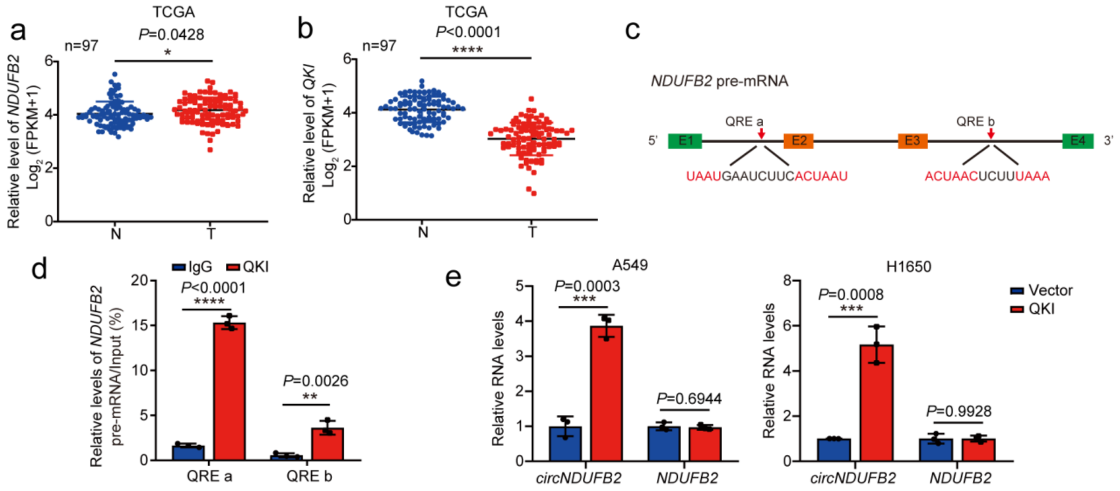


Supplementary Figure 2

QKI promotes *circNDUFB2* formation in NSCLC.

(a-b) mRNA levels of *NDUFB2* (a) and *QKI* (b) for patients with NSCLC in TCGA cohorts. Data are presented as mean ± s.d. P values are calculated by paired two-sided t-test in a-b. n=97 biologically independent paired tissues of NSCLC. (c) A sketch map for potential QRE in the *NDUFB2* pre-mRNA. QRE a and QRE b, two potential QKI response elements in the introns flanking exon 2 and exon 3 of *NDUFB2* pre-mRNA, respectively. (d) Analysis for the *NDUFB2* pre-mRNA enrichment, relative to input. RIP assay was performed using QKI antibody in A549 cells. n=3 biologically independent samples. (e) Expression levels of *circNDUFB2* and *NDUFB2* in A549 and H1650 cells with QKI overexpression. n=3 biologically independent samples. Data are presented as mean ±s.d. P values are calculated by unpaired two-sided t-test in d-e.

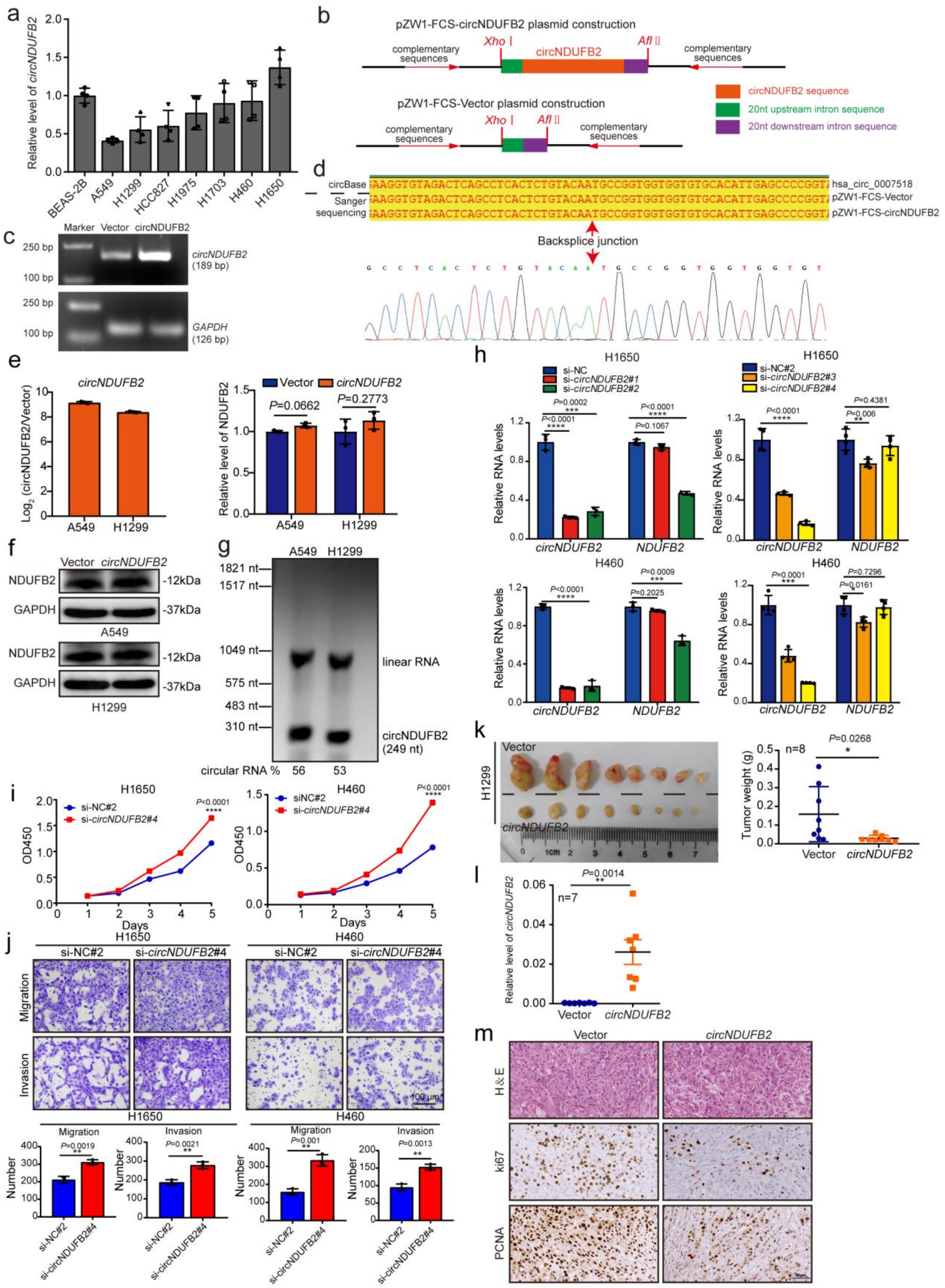


Supplementary Figure 3

Inhibitory effects of *circNDUFB2* on proliferation for NSCLC cells.

(a) Expression levels of *circNDUFB2* in one normal cell line (a human bronchial epithelial cell line BEAS-2B) and seven NSCLC cell lines, relative to BEAS-2B. n=4 biologically independent

samples. (b) A sketch map for plasmid construction of *circNDUFB2* overexpression. (c) PCR analysis for *circNDUFB2* in A549 cells with or without *circNDUFB2* overexpression. (d) Sanger sequencing analysis for PCR products in (c). (e) Expression levels of *circNDUFB2* and *NDUFB2* in NSCLC cells with *circNDUFB2* overexpression. n=3 biologically independent samples. (f) Protein levels of NDUFB2 in NSCLC cells with *circNDUFB2* overexpression. (g) Northern blotting analysis for RNA from A549 cells and H1299 cells transfected with *circNDUFB2* overexpression plasmid. The relative abundance of circular RNA and linear RNA was determined by using the ImageJ program. (h) Expression levels of *circNDUFB2* and *NDUFB2* in NSCLC cells with *circNDUFB2* knockdown. n=3, 3, 4, 4 biologically independent samples, respectively. (i) Cell proliferation assays for NSCLC cells with *circNDUFB2* knockdown. n=5 biologically independent samples. (j) Migration and invasion assays for NSCLC cells with *circNDUFB2* knockdown. n=3 biologically independent samples. Scale bar=100 μm. (k) The weight of subcutaneous xenograft tumors (n=8 mice per group). (l) Expression levels of *circNDUFB2* in subcutaneous xenograft tumors derived from A549 cells. (m) H&E staining and immunohistochemical staining for cell proliferation marker in subcutaneous xenograft tumors derived from A549 cells, scale bar=50 μm. Data are presented as mean ± s.d. P values are calculated by unpaired two-sided t-test in a, e and h-l. Two independent experiments were carried out with similar results in c, f-g and m.

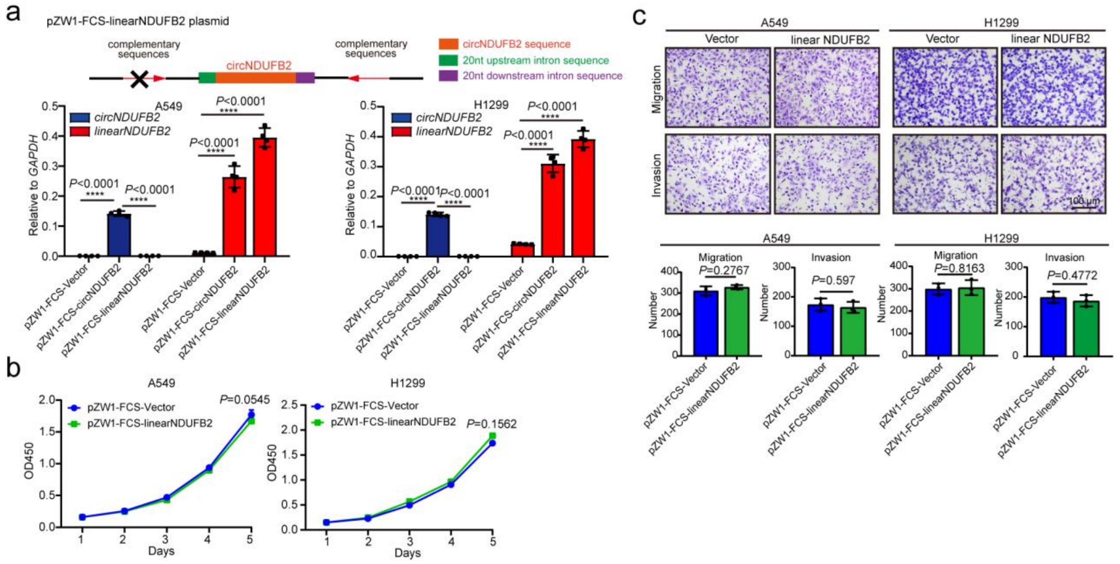


Supplementary Figure 4

Linear-NDUFB2 does not affect NSCLC progression.

(a) Top: A sketch map for pZW1-FCS-linearNDUFB2 plasmid construction. Bottom: qRT-PCR showed pZW1-FCS-circNDUFB2 plasmid produced both *circNDUFB2* and linear-*NDUFB2*, whereas pZW1-FCS-linearNDUFB2 plasmid only produced linear-*NDUFB2*. n=4 biologically independent samples. (b) Cell proliferation assays for NSCLC cells with linear-*NDUFB2* overexpression. n=5 biologically independent samples. (c) Migration and invasion assays for NSCLC cells with linear-*NDUFB2* overexpression. n=3 biologically independent samples. Scale bar=100 μm. Data are presented as mean ± s.d. P values are calculated by unpaired two-sided t-test in a-c.

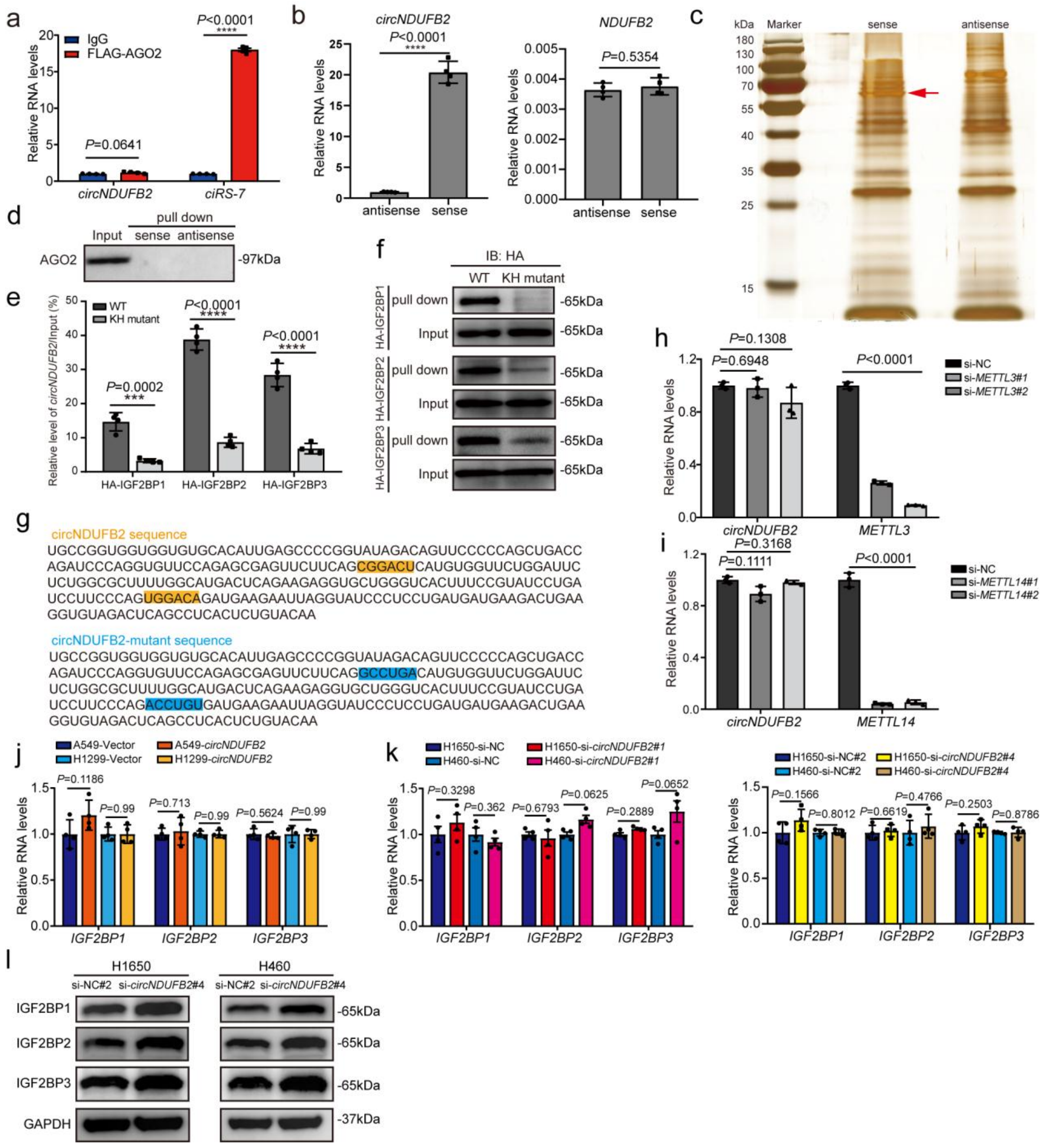


Supplementary Figure 5

*circNDUFB2* binds with IGF2BPs.

(a) Analysis for *circNDUFB2* and *ciRS-7* enrichment, relative to IgG. RIP assays were performed using FLAG antibody in A549 cells transfected with FLAG-AGO2 plasmid. n=4 biologically independent samples. (b) Analysis for *NDUFB2* and *circNDUFB2* enrichment. RNA pull down assays were performed using biotinylated sense probe which targets the *circNDUFB2* backsplice junction region. n=4 biologically independent samples. (c) Biotinylated sense probe and antisense

probe were incubated with A549 total cell lysates for RNA pull down assays. After silver staining, the sense-specific band at about 65kDa (red arrow) was excised and analysed using mass spectrometry. (d) RNA pull down showed that *circNDUFB2* didn't interact with AGO2. (e) Analysis for the *circNDUFB2* enrichment, relative to input. RIP assay was performed using HA antibody in the indicated cells. n=4 biologically independent samples. (f) Binding of *circNDUFB2* to IGF2BPs or KH domain mutant IGF2BPs in vitro. (g) Sequences of *circNDUFB2* and *circNDUFB2*-mutant. For the *circNDUFB2*-mutant plasmid, 'CGGACU' was replaced with 'GCCUGA', and 'UGGACA' was replaced with 'ACCUGU', respectively. (h) Expression levels of *METTL3* and *circNDUFB2* in A549 cells with METTL3 knockdown. n=3 biologically independent samples. (i) Expression levels of *METTL14* and *circNDUFB2* in A549 cells with METTL14 knockdown. n=3 biologically independent samples. (j-k) mRNA levels of *IGF2BPs* in NSCLC cells with *circNDUFB2* overexpression (j) or knockdown (k). n=4 biologically independent samples. (l) Protein levels of IGF2BPs in NSCLC cells with *circNDUFB2* knockdown. Data are presented as mean ± s.d. P values are calculated by unpaired two-sided t-test in a-b, e and h-k. Two independent experiments were carried out with similar results in c-d, f and l.

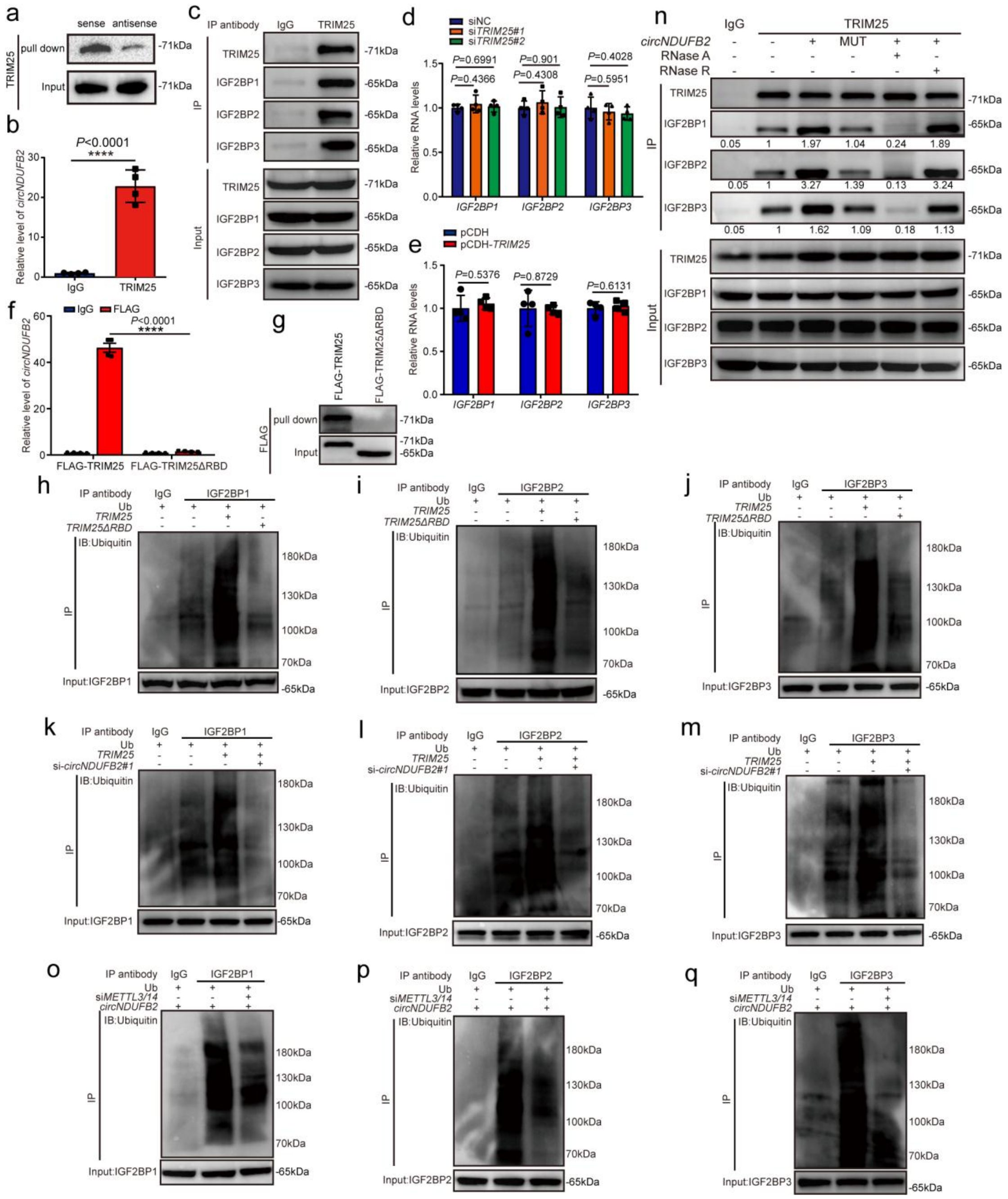

Supplementary Figure 6

TRIM25 promotes ubiquitination and degradation of IGF2BPs in a *circNDUFB2*-dependent manner.

(a) Binding of *circNDUFB2* with TRIM25 in H1299 cells. (b) Analysis for *circNDUFB2* enrichment, relative to IgG. RIP assay was performed using TRIM25 antibody in A549 cells. n=4 biologically independent samples. (c) Co-immunoprecipitation (Co-IP) showed the binding of

TRIM25 with IGF2BPs in A549 cells. (d-e) mRNA levels of *IGF2BPs* in A549 cells with TRIM25 knockdown (d) or overexpression (e), respectively. n=4 biologically independent samples. (f) Analysis for *circNDUFB2* enrichment, relative to IgG. RIP assays were performed using FLAG antibody in A549 cells. n=4 biologically independent samples. (g) RNA pull down assays were performed using biotinylated sense probe for *circNDUFB2* in A549 cells. (h-j) Ubiquitination modification of IGF2BPs in A549 cells transfected with FLAG-TRIM25 or FLAG-TRIM25ΔRBD plasmids. (k-m) Ubiquitination modification of IGF2BPs in A549 cells with TRIM25 overexpression and *circNDUFB2* knockdown. (n) Co-IP showed the binding of TRIM25 with IGF2BP proteins. (o-q) Ubiquitination modification of IGF2BPs in A549 cells with METTL3/14 knockdown. Ub, ubiquitin. Data are presented as mean ± s.d. P values are calculated by unpaired two-sided t-test in b and d-f. Two independent experiments were carried out with similar results in a, c, g and h-q.

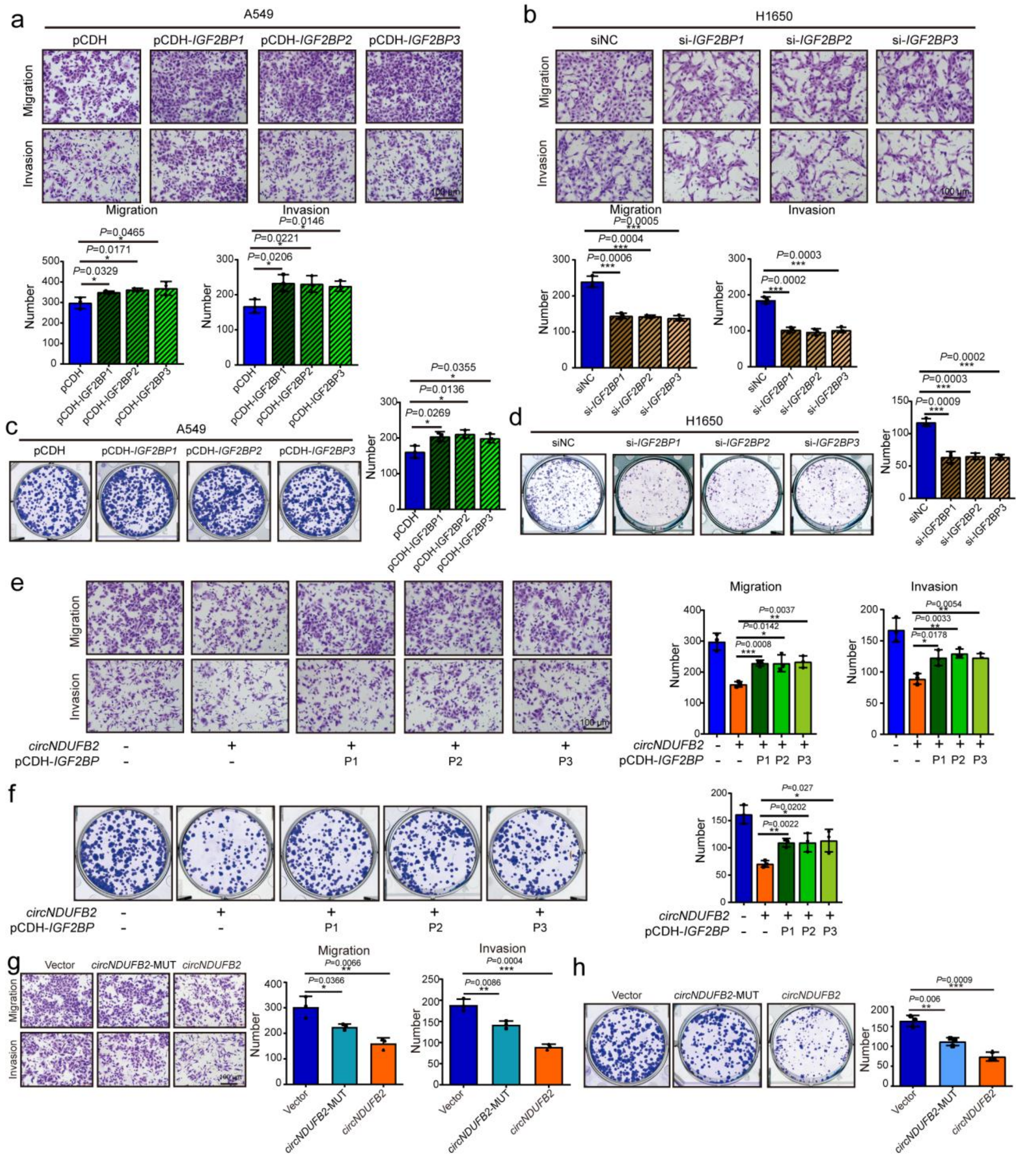

Supplementary Figure 7

IGF2BPs mediate inhibitory effects of *circNDUFB2* on NSCLC progression.

(a-b) Migration and invasion assays for NSCLC cells with IGF2BPs overexpression or knockdown. n=3 biologically independent samples. Scale bar=100 μm. (c-d) Colony formation assays for NSCLC cells with IGF2BPs overexpression or knockdown. n=3 biologically independent samples. (e) Migration and invasion assays showed the restoring effects of IGF2BPs on A549 cells with *circNDUFB2* overexpression. P1, IGF2BP1; P2, IGF2BP2; P3, IGF2BP3. n=3

biologically independent samples. Scale bar=100 μm. (f) Colony formation assays showed the restoring effects of IGF2BPs on A549 cells with *circNDUFB2* overexpression. P1, IGF2BP1; P2, IGF2BP2; P3, IGF2BP3. n=3 biologically independent samples. (g) Migration and invasion assays for A549 cells with overexpression of *circNDUFB2* or *circNDUFB2*-mutant. n=3 biologically independent samples. Scale bar=100 μm. (h) Colony formation assays for A549 cells with overexpression of *circNDUFB2* or *circNDUFB2*-mutant. n=3 biologically independent samples. Data are presented as mean ± s.d. P values are calculated by unpaired two-sided t-test in a-h. Two independent experiments were carried out with similar results in a, b, e and g.

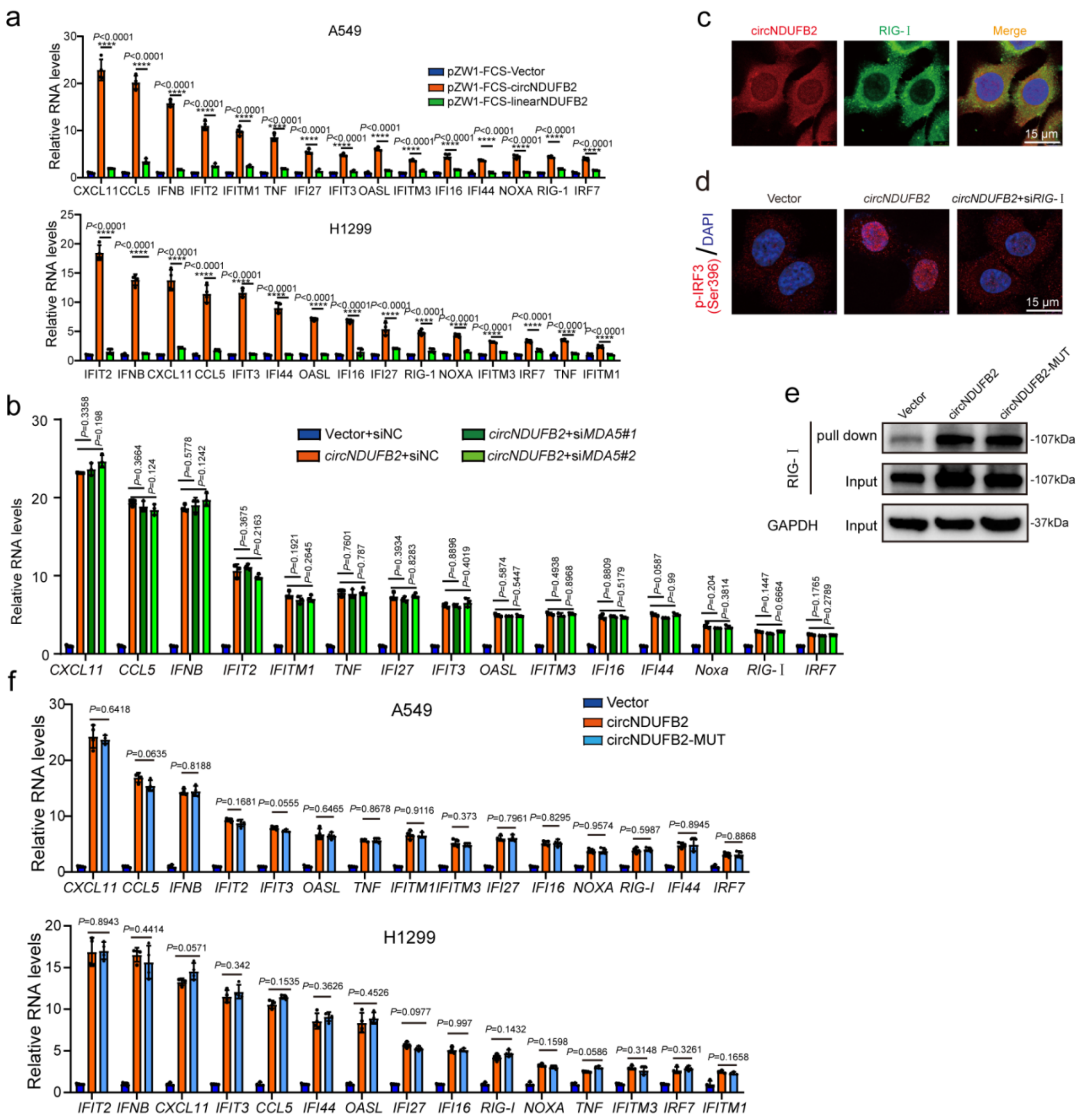

Supplementary Figure 8

*circNDUFB2* binds with RIG-I.

(a) Expression levels of indicated mRNA in NSCLC cells with *circNDUFB2* or linear-*NDUFB2* overexpression. n=4 biologically independent samples. (b) Fold change of indicated mRNAs in A549 cells with or without MDA5 knockdown. n=3 biologically independent samples. (c) RNA FISH-immunofluorescence showed the co-localization of *circNDUFB2* (red) with RIG-I (green) in A549 cells. Scale bar=15 μm. (d) Immunofluorescence detected the localization of p-IRF3 in A549 cells. Scale bar=15 μm. (e) RNA pull down assay was performed using biotinylated sense

probe for *circNDUFB2* in A549 cells with *circNDUFB2* or *circNDUFB2*-MUT overexpression. (f) Expression levels of indicated mRNAs in NSCLC cells with *circNDUFB2* or *circNDUFB2* mutant overexpression. n=4 biologically independent samples. Data are presented as mean ± s.d. P values are calculated by unpaired two-sided t-test in a-b and f. Two independent experiments were carried out with similar results in c-e.

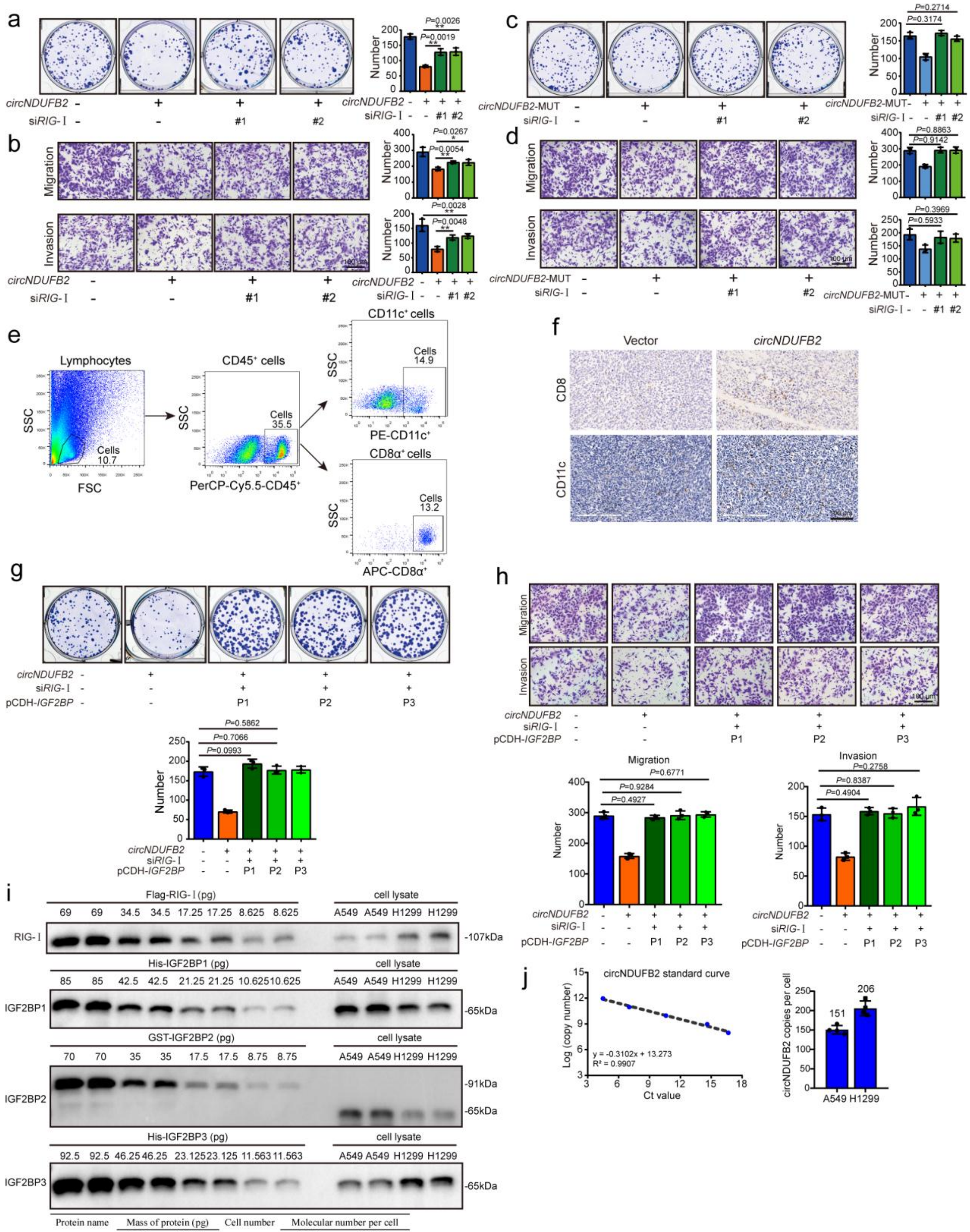


| Protein name | Mass of protein (pg) | | Cell number | Molecular number per cell | |
|---|---|---|---|---|---|
| | A549 | H1299 | | A549 | H1299 |
| RIG-I | 1.776 | 10.132 | 200000 | 50 | 286 |
| IGF2BP1 | 53.88 | 37.57 | 500000 | 1021 | 712 |
| IGF2BP2 | 19.51 | 13.51 | 200000 | 888 | 615 |
| IGF2BP3 | 16.53 | 27.27 | 500000 | 312 | 515 |

Supplementary Figure 9

Immune responses of *circNDUFB2* mediated by RIG-I inhibits tumor progression.

(a-b) Colony formation assays and migration assays showed the restoring effects of RIG-I on A549 cells with *circNDUFB2* overexpression. n=3 biologically independent samples. Scale

bar=100 μm. (c-d) Colony formation assays and migration assays showed the restoring effects of RIG-I on A549 cells with *circNDUFB2*-MUT overexpression. n=3 biologically independent samples. Scale bar=100 μm. (e) Gating strategy and representative flow cytometry plots for the assessment of $CD45^+$, $CD8\alpha^+$ and $CD11c^+$ cells in *circNDUFB2* overexpression and control LLC1 tumors. (f) IHC staining for CD8 and CD11c in subcutaneous xenograft tumors, scale bar=100 μm. (g-h) Colony formation assays and migration assays showed the restoring effects of IGF2BPs and RIG-I on A549 cells with *circNDUFB2* overexpression. n=3 biologically independent samples. Scale bar=100 μm. (i) Measurement of protein molecular number per cell. Top: western blot analysis for purified recombinant protein and cell lysate corresponding to a known number of NSCLC cells. Bottom: quantification summarized in table. Two independent experiments were carried out with similar results. (j) Copy number of *circNDUFB2* in A549 cells and H1299 cells. Left: the linear relationship between the log *circNDUFB2* copy number and its Ct value by qRT-PCR. Right: the average *circNDUFB2* copies per A549 cell and H1299 cell. n=4 biologically independent samples. P1, IGF2BP1; P2, IGF2BP2; P3, IGF2BP3; Data are presented as mean ± s.d. P values are calculated by unpaired two-sided t-test in a-d, g-h and j.

Supplementary Table 1

Associations between *circNDUFB2* and clinicopathologic features in 52 patients with NSCLC

| Clinicopathological features | Number of patients | *circNDUFB2* | | P value |
|---|---|---|---|---|
| | | Low | High | |
| Age (y) | | | | 0.1259 |
| <60 | 15 | 5 | 10 | |
| ≥60 | 37 | 21 | 16 | |
| Gender | | | | 0.0922 |
| Male | 30 | 18 | 12 | |
| Female | 22 | 8 | 14 | |
| Smoke | | | | 0.0714 |
| Smoker | 16 | 11 | 5 | |
| Nonsmoker | 36 | 15 | 21 | |
| Tumor size (cm) | | | | 0.0337[a] |
| <3 | 16 | 4 | 12 | |
| ≥3 | 36 | 22 | 14 | |
| Lymph node metastasis | | | | 0.0226[a] |
| No | 20 | 6 | 14 | |
| Yes | 32 | 20 | 12 | |
| Stage | | | | 0.011[a] |
| Ⅰ+Ⅱ | 31 | 11 | 20 | |
| Ⅲ+Ⅳ | 21 | 15 | 6 | |

The associations between *circNDUFB2* and clinicopathologic features in patients with NSCLC were analyzed. NSCLC, non-small cell lung cancer. The median expression level was used as the cutoff.

[a]P<0.05, which was considered as a significant difference.

Supplementary Table 2

Clinicopathological characteristics of 55 NSCLC patients used in this study

| Cases used for Arraystar Human circRNAs Array analysis (n=3) | | | | | | |
|---|---|---|---|---|---|---|
| Case | Age | Gender | Smoke | Tumor Size (cm) | Lymph node metastasis | Stage |
| 1 | 64 | Male | YES | 3.5*2.5*3 | YES | ⅠB |
| 2 | 56 | Female | NO | 3.5*3*2.5 | YES | ⅡA |
| 3 | 53 | female | NO | 4*3*2.5 | YES | ⅢA |

| Cases used for qRT-PCR validation (n=52) | | | | | |
|---|---|---|---|---|---|
| Age (<60:≥60) | Gender (Male:Female) | Smoke (NO:YES) | Tumor size, cm (<3:≥3) | Lymph node metastasis (NO:YES) | Stage (Ⅰ+Ⅱ:Ⅲ+Ⅳ) |
| 15:37 | 30:22 | 36:16 | 14:38 | 19:33 | 31:21 |

Supplementary Table 3

Mass spectrometry identification of proteins pulled down at about 65kDa

| Gene name | Protein_score | Protein_coverage | MW [kDa] |
|---|---|---|---|
| *IGF2BP2* | 566 | 23.2 | 66.2 |
| *IGF2BP1* | 211 | 14.7 | 63.8 |
| *IGF2BP3* | 175 | 10.5 | 64 |
| *COIL* | 142 | 12.7 | 63.3 |
| *HNRNPLL* | 118 | 10.7 | 60.9 |
| *NOP56* | 87 | 6.2 | 66.4 |
| *CLK2* | 75 | 6 | 60.5 |
| *KPNA1* | 46 | 3.2 | 60.9 |
| *STAU1* | 40 | 2.9 | 63.4 |
| *PUF60* | 35 | 1.8 | 60 |
| *CSTF2* | 34 | 1 | 61 |
| *DDX56* | 32 | 0.9 | 62 |
| *HIRIP3* | 32 | 0.9 | 62.3 |
| *KPNA6* | 30 | 3 | 60.7 |
| *CSRNP1* | 30 | 4.1 | 64.8 |
| *INSM2* | 26 | 0.9 | 60.5 |
| *TCP1* | 26 | 1.8 | 60.8 |
| *DDX28* | 25 | 1.7 | 59.8 |
| *UGT1A7* | 25 | 0.9 | 60.6 |
| *RELA* | 24 | 1.1 | 60.7 |
| *CRMP1* | 24 | 0.9 | 62.5 |
| *CCDC102A* | 24 | 1.1 | 62.8 |
| *CPNE3* | 23 | 1.7 | 60.9 |
| *ZNF18* | 23 | 1.3 | 63.2 |
| *TMEM200C* | 23 | 1 | 64.3 |
| *PANK2* | 21 | 0.9 | 63.3 |

Supplementary Table 4

Antibodies used in this study

| Antibodies | Source | Identifier |
|---|---|---|
| Anti-AGO2 | Abcam | Cat#ab186733 |
| Anti-CD8 | Abcam | Cat#ab22378 |
| Anti-IFNβ | Abcam | Cat#ab176343 |
| Anti-ki67 | Abcam | Cat#ab16667 |
| Anti-PANA | Abcam | Cat#ab29 |
| Anti-TRIM25 | Abcam | Cat#ab88669 |
| Anti-GAPDH | Proteintech | Cat#60004-1-Ig |
| Anti-HA | Proteintech | Cat#51064-2-AP |
| Anti-HECTD3 | Proteintech | Cat#11487-1-AP |
| Anti-IGF2BP1 | Proteintech | Cat#22803-1-AP |
| Anti-IGF2BP2 | Proteintech | Cat#11601-1-AP |
| Anti-IGF2BP3 | Proteintech | Cat#14642-1-AP |
| Anti-IRF3 | Proteintech | Cat#11312-1-AP |
| Anti-IRF7 | Proteintech | Cat#22392-1-AP |
| Anti-NDUFB2 | Proteintech | Cat#17614-1-AP |
| Anti-RIG-I | Proteintech | Cat#20566-1-AP |
| Anti-TNF | Proteintech | Cat#60291-1-Ig |
| Anti-TRIM25 | Proteintech | Cat#12573-1-AP |
| Anti-Ubiquitin | Proteintech | Cat#10201-2-AP |
| Anti-CD11C | Cell Signaling Technology | Cat#97585 |
| Anti-P65 | Cell Signaling Technology | Cat#8242 |
| Anti-p-P65 (Ser536) | Cell Signaling Technology | Cat#3033 |
| Anti-p-IRF3 (Ser396) | Cell Signaling Technology | Cat#29047 |
| Anti-p-STAT1 (Tyr701) | Cell Signaling Technology | Cat#9167 |
| Anti-p-STAT2 (Tyr690) | Cell Signaling Technology | Cat #88410 |
| Anti-rabbit IgG (H+L), F(ab')2 Fragment (Alexa Fluor® 488 Conjugate) | Cell Signaling Technology | Cat#4412 |
| Anti-rabbit IgG (H+L), F(ab')2 Fragment (Alexa Fluor® 594 Conjugate) | Cell Signaling Technology | Cat#8889 |
| Anti-mouse IgG (H+L), F(ab')2 Fragment (Alexa Fluor® 488 Conjugate) | Cell Signaling Technology | Cat#4408 |
| Anti-CD8α | Biolegend | Cat#100712 |
| Anti-CD11C | Biolegend | Cat#117308 |
| Anti-CD45 | Invitrogen | Cat#45-0451-82 |
| Anti-QKI | BETHYL | Cat#A300-183A |
| Anti-Flag | Sigma | Cat#F1804 |
| Anti-rabbit-IgG-HRP | Bioworld | Cat#BS13278 |
| Anti-mouse-IgG-HRP | Bioworld | Cat#BS12478 |
