# Supplementary material for: circNDUFB2 inhibits non-small cell lung cancer progression via destabilizing IGF2BPs and activating anti-tumor immunity
Source: Nat Commun. 2021 Jan 12;12:295. doi: 10.1038/s41467-020-20527-z (PMC7804955; doi:10.1038/s41467-020-20527-z)
Supplement: Supplementary file 3 — Reporting Summary [file 41467_2020_20527_MOESM3_ESM.pdf]

nature research

Corresponding author(s): Wenxin Qin

Last updated by author(s): November 30, 2020

Reporting Summary

Nature Research wishes to improve the reproducibility of the work that we publish. This form provides structure for consistency and transparency in reporting. For further information on Nature Research policies, see our Editorial Policies and the Editorial Policy Checklist.

Statistics

For all statistical analyses, confirm that the following items are present in the figure legend, table legend, main text, or Methods section.

n/a	Confirmed	
☐	☒	The exact sample size (*n*) for each experimental group/condition, given as a discrete number and unit of measurement
☐	☒	A statement on whether measurements were taken from distinct samples or whether the same sample was measured repeatedly
☐	☒	The statistical test(s) used AND whether they are one- or two-sided<br>*Only common tests should be described solely by name; describe more complex techniques in the Methods section.*
☒	☐	A description of all covariates tested
☒	☐	A description of any assumptions or corrections, such as tests of normality and adjustment for multiple comparisons
☐	☒	A full description of the statistical parameters including central tendency (e.g. means) or other basic estimates (e.g. regression coefficient) AND variation (e.g. standard deviation) or associated estimates of uncertainty (e.g. confidence intervals)
☐	☒	For null hypothesis testing, the test statistic (e.g. *F*, *t*, *r*) with confidence intervals, effect sizes, degrees of freedom and *P* value noted<br>*Give P values as exact values whenever suitable.*
☒	☐	For Bayesian analysis, information on the choice of priors and Markov chain Monte Carlo settings
☒	☐	For hierarchical and complex designs, identification of the appropriate level for tests and full reporting of outcomes
☐	☒	Estimates of effect sizes (e.g. Cohen's *d*, Pearson's *r*), indicating how they were calculated

Our web collection on statistics for biologists contains articles on many of the points above.

Software and code

Policy information about availability of computer code

Data collection	qRT-PCR data were collected by the ABI 7500 Fast Dx instruments' Sequence Detection Software v2.0.5; Western blot images were captured using Bio-Rad Image Lab software v 5.2.1; For immunofluorescence and FISH, fluorescent images were acquired using OLYMPUS FV1000 confocal microscopy; Flow cytometry was performed on an LSRII (BD Biosciences) and data were analysed using FlowJo software v 10.4.2 (FlowJo).
Data analysis	Microsoft Excel 2007 and Graph Pad Prism 8 for statistical analysis and graph plotting.

For manuscripts utilizing custom algorithms or software that are central to the research but not yet described in published literature, software must be made available to editors and reviewers. We strongly encourage code deposition in a community repository (e.g. GitHub). See the Nature Research guidelines for submitting code & software for further information.

Data

Policy information about availability of data

All manuscripts must include a data availability statement. This statement should provide the following information, where applicable:

- Accession codes, unique identifiers, or web links for publicly available datasets
- A list of figures that have associated raw data
- A description of any restrictions on data availability

Microarray data are accessible at the Gene Expression Omnibus (GEO) under accession number: GSE158695, https://www.ncbi.nlm.nih.gov/geo/query/acc.cgi?acc=GSE158695. RNA-seq data are accessible at the GEO under accession number: GSE156607, https://www.ncbi.nlm.nih.gov/geo/query/acc.cgi?acc=GSE156607. The source data underlying Figs. 1b, e-g, 2c-h, 3b, e, 5d-g, 6a,b,e, h-j and Supplementary Figs. 1a-d, 2a, b, d, e, 3a, e, h, j-l, 4a-c, 5a, b, e, h-k, 6b, d-f, 7a-h, 8a, b, f and 9a-d, g, h, j are provided as a Source Data file. Unprocessed gel images for Figs. 1d, 3a, d, f-j, 4a, c, e-h, 6c, d, f and Supplementary Figs. 3c, f, g, 5d, f, l, 6a, c, g, h-q, 8e and 9i are provided as a Source Data file. Other data that support the findings of this study are available from the corresponding authors on reasonable

request.

Field-specific reporting

Please select the one below that is the best fit for your research. If you are not sure, read the appropriate sections before making your selection.

- [x] Life sciences
- [ ] Behavioural & social sciences
- [ ] Ecological, evolutionary & environmental sciences

For a reference copy of the document with all sections, see nature.com/documents/nr-reporting-summary-flat.pdf

Life sciences study design

All studies must disclose on these points even when the disclosure is negative.

Sample size	In this study, 3 paired samples of NSCLC patients were used for Microarray analysis and 52 paired samples of NSCLC patients were used for qRT-PCR validation. In general, no statistical methods were used to predetermine sample size. For the animal experiments, sample size was determined based on previous experience with models of NSCLC and literature reports. For human samples, samples were collected until we felt the sample size was sufficient to give reliable estimates. For cell and biochemical data, we aimed to collect data from three biological replicates when possible.
Data exclusions	No data have been excluded from the analysis.
Replication	All in vitro experiments were repeated at least two independent times and all attempts at replication were successful. For xenograft experiments, five or more biologically independent tumors were used. All in vivo studies were performed two times and the data were reproducible between each study.
Randomization	For in vitro study, cells were based on gain or loss of function experiments with appropriate controls. Cells were seeded identically at the onset of the experiments and randomized into the various treatment groups prior to the beginning of treatment protocols. For in vivo studies, mice were allocated into experimental groups randomly and we did not control for covariates in the animal experiments because all mice were the same age and sex and were purchased from the same supplier.
Blinding	For the other experiments on human samples, mice and cell lines, blinding was not performed due to feasibility.

Reporting for specific materials, systems and methods

We require information from authors about some types of materials, experimental systems and methods used in many studies. Here, indicate whether each material, system or method listed is relevant to your study. If you are not sure if a list item applies to your research, read the appropriate section before selecting a response.

Materials & experimental systems

n/a	Involved in the study	
[ ]	[x]	Antibodies
[ ]	[x]	Eukaryotic cell lines
[x]	[ ]	Palaeontology and archaeology
[ ]	[x]	Animals and other organisms
[ ]	[x]	Human research participants
[x]	[ ]	Clinical data
[x]	[ ]	Dual use research of concern

Methods

n/a	Involved in the study	
[x]	[ ]	ChIP-seq
[ ]	[x]	Flow cytometry
[x]	[ ]	MRI-based neuroimaging

Antibodies

Antibodies used	Information of antibodies used in this study were provided in supplementary Table 4. The dilution used for each antibody described in the methods section of the manuscript.
Validation	All commercially available antibodies were validated by vendors. Validation statements are provided on the manufacture's website. We examined primary antibodies according to manuals, and got similar results with validation results on manufacturer's website or relevant citations.

Eukaryotic cell lines

Policy information about cell lines

Cell line source(s)	Human cell lines (A549, H1299, HCC827, H1975, H1703, H460, H1650 and BEAS-2B) and murine lung carcinoma cell line (LLC1) used in this study were described in Methods section. They were acquired from ATCC.

April 2020

Authentication	Cell lines were obtained from original sources and were not further authenticated.
Mycoplasma contamination	All cell lines used in the study were tested negative for mycoplasma contamination.
Commonly misidentified lines (See ICLAC register)	No commonly misidentified cell lines were used in the study.

Animals and other organisms

Policy information about studies involving animals; ARRIVE guidelines recommended for reporting animal research

Laboratory animals	Female BALB/c nude mice and C57BL/6 mice (six-eight weeks old) were maintained under SPF conditions in a controlled environment of 20-22 ℃, with a 12/12 h light/dark cycle, 50-70% humidity, and food and water provided ad libitum.
Wild animals	No wild animals were used in the study.
Field-collected samples	No field collected samples were used in the study.
Ethics oversight	Mouse experiments were approved by the Shanghai Medical Experimental Animal Care Commission.

Note that full information on the approval of the study protocol must also be provided in the manuscript.

Human research participants

Policy information about studies involving human research participants

Population characteristics	In total, 55 paired primary NSCLC tumorous tissues (T) and adjacent nontumorous tissues (N) were collected from patients who had undergone surgery at Shanghai Chest Hospital (Shanghai, China). The detailed clinicopathological characteristics were described in Supplementary Tables 1, 2. All tissue specimens were collected from July 2013 to September 2014 with the consent of patients and approved by Ethics Committee of the Shanghai Chest Hospital.
Recruitment	Participants were recruited from the pool of patients at Shanghai Chest Hospital. No selection bias was observed.
Ethics oversight	All tissue specimens were collected from July 2013 to September 2014 with the consent of patients and approved by Ethics Committee of the Shanghai Chest Hospital. Patient informed consent form was properly signed before recruitment for the study.

Note that full information on the approval of the study protocol must also be provided in the manuscript.

Flow Cytometry

Plots

Confirm that:

- [x] The axis labels state the marker and fluorochrome used (e.g. CD4-FITC).
- [x] The axis scales are clearly visible. Include numbers along axes only for bottom left plot of group (a 'group' is an analysis of identical markers).
- [x] All plots are contour plots with outliers or pseudocolor plots.
- [x] A numerical value for number of cells or percentage (with statistics) is provided.

Methodology

Sample preparation	Tumor-infiltrating lymphocytes were stained directly from single-cell preparations of ex vivo tumors as described in the materials and methods.
Instrument	BD LSR Fortessa
Software	BD DIVA software used for collection, FlowJo software used for analysis.
Cell population abundance	No cell sorting was performed in this manuscript.
Gating strategy	All gates were set based on FMO (full-minus one) stains and isotype control antibodies after appropriate compensation using single-stained compensation controls.

- [x] Tick this box to confirm that a figure exemplifying the gating strategy is provided in the Supplementary Information.
