# Supplementary material for: circNDUFB2 inhibits non-small cell lung cancer progression via destabilizing IGF2BPs and activating anti-tumor immunity
Source: Nat Commun. 2021 Jan 12;12:295. doi: 10.1038/s41467-020-20527-z (PMC7804955; doi:10.1038/s41467-020-20527-z)
Supplement: Supplementary file 4 — Description of Additional Supplementary Files [file 41467_2020_20527_MOESM4_ESM.pdf]

## Description of Additional Supplementary Files

Supplementary Data 1. 109 significantly dysregulated circRNAs

Supplementary Data 2. Mass spectrometry identification of proteins pulled down by circNDUFB2 sense probe

Supplementary Data 3. RNA-seq analysis of the gene expression profile affected by circNDUFB2 overexpression

Supplementary Data 4. Sequences of primer, siRNA and RNA pull down probe used in this study
